# Diagnostic and Management of Emphysematous Hepatitis with Emphasis on Biopathology

**DOI:** 10.3390/microorganisms11092137

**Published:** 2023-08-23

**Authors:** Déborah Porez, Hatem Kallel, Succes Dobian, Timotée Gerbert-Ferrendier, Mathieu Nacher, Félix Djossou, Magalie Demar, Hakim Amroun, Magaly Zappa, Kinan Drak Alsibai

**Affiliations:** 1 Unité des Maladies Infectieuses Tropicales (UMIT), Cayenne Hospital Centre, F-97306 Cayenne, France; deborah.porez@ch-cayenne.fr (D.P.); felix.djossou@ch-cayenne.fr (F.D.); 2 Réanimation Polyvalente, Pôle Urgences-Soins Critiques, Cayenne Hospital Centre, F-97306 Cayenne, France; hatem.kallel@ch-cayenne.fr; 3 Service d’Imagerie Médicale, Cayenne Hospital Centre, F-97306 Cayenne, France; succes.dobian@ch-cayenne.fr (S.D.); magaly.zappa@ch-cayenne.fr (M.Z.); 4 Department of Pathology, Cayenne Hospital Centre, F-97306 Cayenne, France; timotee.gerbertferrendier@gmail.com; 5 Amazin PopHealth, Département de Recherche et d’Innovation en Santé Publique (DRISP), Inserm Centre d’Investigation Clinique (CIC 1424), Cayenne Hospital Centre Andrée Rosemon, F-97300 Cayenne, France; mathieu.nacher@ch-cayenne.fr; 6 Department of Surgery, Cayenne Hospital Centre, F-97306 Cayenne, France; magalie.demar@ch-cayenne.fr; 7 Laboratoire Polyvalent, Cayenne Hospital Centre, F-97306 Cayenne, France; hakim.amroun@ch-cayenne.fr; 8 Centre of Biological Resources (CRB Amazonie), Cayenne Hospital Centre, F-97306 Cayenne, France

**Keywords:** emphysematous hepatitis, *Klebsiella pneumoniae*, *Clostridium perfingens* liver infectious disease, biopathology

## Abstract

Emphysematous hepatitis is a rare infectious disease, which corresponds to the presence of gas in the liver, without collection and after exclusion of vascular origin. This entity belongs to the group of emphysematous infections, whose physiopathology seems to be linked to the presence of unbalanced diabetes and to bacterial fermentation, leading to the production of gas within the liver parenchyma. Very few cases of emphysematous hepatitis have been described in the literature, and most of them had a rapidly fatal course. In this manuscript, we report the case of a 59-year-old man with emphysematous hepatitis due to wild-type *Klebsiella pneumoniae* that was successfully managed by surgery, and we perform a review of the literature to describe the clinical and biopathological aspects of this rare hepatic disease. Our manuscript underlines the need to perform biological and histopathological sampling to better understand the pathophysiology of this rare entity. The causes and mechanisms of emphysematous hepatitis, which seem to be multiple, lead us to believe that it is a syndrome rather than a simple infectious disease.

## 1. Introduction

Emphysematous hepatitis is a rare and recent entity, first described in 2002 [1]. It corresponds to the presence of gas in the liver, without collection and after exclusion of vascular origin. This entity belongs to the group of emphysematous infections whose physiopathology seems to be linked to the presence of unbalanced diabetes and to bacterial fermentation, leading to the production of gas [2].

Emphysematous hepatitis must be distinguished from other etiologies associated with the presence of air in the liver. The air is initially present in the liver parenchyma and not in the vascular, biliary or lymphatic ducts, but may secondarily disseminate to these structures. Emphysematous hepatitis differs from gas-secreting pyogenic abscesses by the absence of wall, pus and collection inside the lesion [3]. Few cases of emphysematous hepatitis have been described in the literature, and most of them had a rapidly fatal course.

We describe here a case of emphysematous hepatitis that was successfully managed by surgery and antimicrobial drugs; then, we perform a literature review to describe the clinical and histopathological aspects of this rare hepatic disease.

## 2. Materials and Methods

### 2.1. Case Report

We report the case of a 59-year-old man who presented to the Emergency Unit of Cayenne Hospital (Cayenne, French Guiana) for fever and worsened general condition. He had a medical history of uncontrolled type 2 diabetes and hypertension. On arrival at the Emergency Unit, the patient was febrile (39.8 °C) and confused, but had no hemodynamic or respiratory failure. His abdomen was soft and painless.

Biological analysis showed a blood glucose level of 42.2 mmol/L (N: 3.88–6.39), ketoacidosis with a pH of 7.26 (N: 7.31–7.42), lactatemia at 2.5 mmol/L (N: 1.1–2.5), and serum bicarbonates at 9 mmol/L (N: 2–30). Leukocytes were 11.7 10^3^/L (N: 4–11) with 70% neutrophils. C-reactive protein (CRP) was 307 mg/L (N: 0–5) with aspartate aminotransferase (AST) at 138 IU/L (N: 0–38) and total bilirubin at 23.8 (N: 0–24) µmol/L. The patient had acute renal failure with a creatinine up to 186 µmol/L (N: 62–107), and had low platelets at 96 × 10^9^/L (N: 150–450).

**Figure 1:**
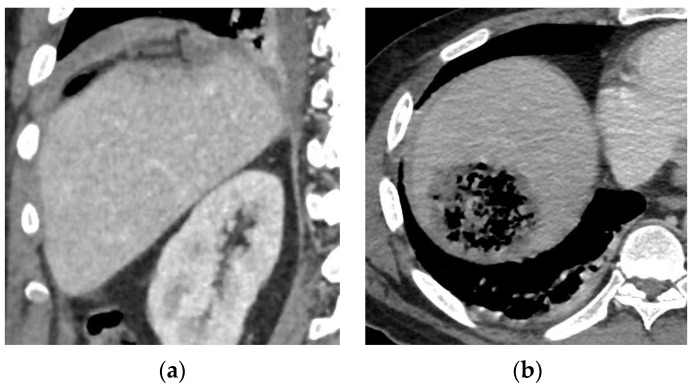
Axial CT scan image after contrast injection showing a poorly limited area containing a little liquid and mainly many gas bubbles, without enhancement wall nor collection (**a**). Coronal CT scan image after contrast injection showing surgical resection area and the Pezzer drain (**b**). Hounsfield scale (HU): 60 HU for liver paranchyma and −1000 HU for air.

We conducted abdominal ultrasounds and then a computed tomography (CT) scan with contrast. It found a poorly limited area in the liver containing multiple gas bubbles without enhancement wall nor collection, located in the right anterior segment dome (segment VIII) measuring 58 × 50 mm associated with moderate left aerobilia (Figure 1). An ultrasound-guided fine needle aspiration (FNA) was performed and sent to the laboratory for microbiological analysis.

The patient was transferred to the Intensive Care Unit to manage the hydroelectrolytic disorders. He was treated with intravenous antibiotics, including ceftriaxone 2 g/24 h and metronidazole 1500 mg/24 h. Ultrasound-guided drainage of the lesion was also performed the day after.

**Figure 2:**
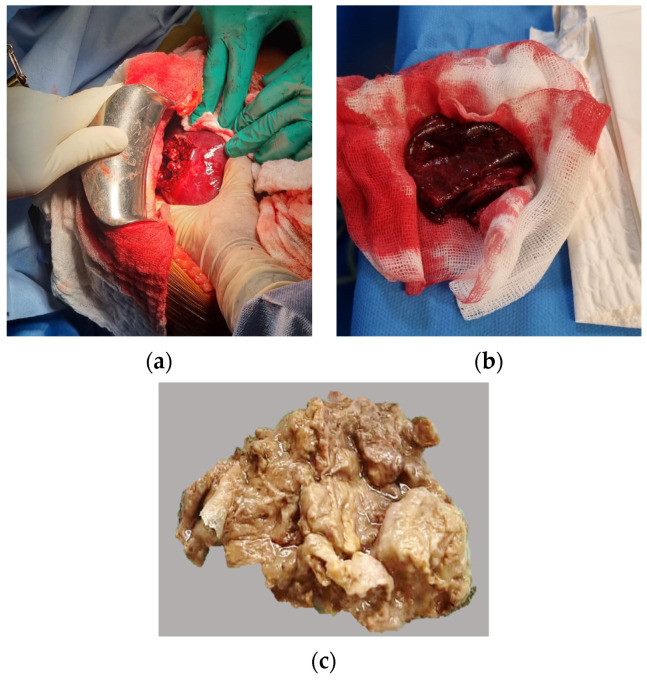
Surgical aspect of the liver specimen (**a**,**b**). Macroscopical image of the internal surface of the surgical specimen fixed in formol (**c**).

Two days after the patient’s arrival in our hospital and in the absence of clear clinical improvement, surgical management was performed by median laparotomy, drainage, and placement of a Pezzer drain in order to decrease the inoculum (Figure 2a,b). A Pezzer drain is a catheter with a swollen and capped end that holds it in place, widely used to drain natural and pathological cavities. Blood cultures, liver ultrasound-guided FNA and surgical samples, as well as cytobacteriological examination of urine (CBEU), were positive for wild-type *Klebsiella pneumoniae*. The string test was in favour of a hyper mucosal phenotype, but we were unable to perform molecular tests to confirm the presence of a hypervirulent phenotype.

**Figure 3:**
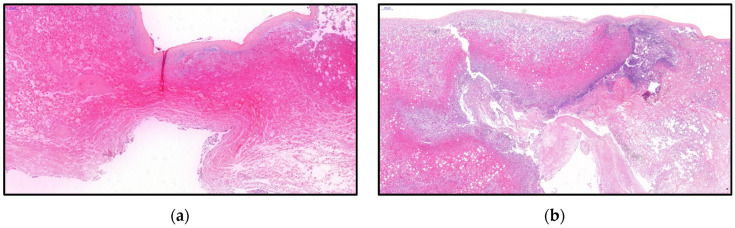
Histopathological features of emphysematous hepatitis from our case report. (**a**) This image shows the emphysematous lesion characterised by an empty space with a roof of completely necrotic liver tissue replaced by a fibrinous and haemorrhagic coating (Haematoxylin and Eosin stain, ×200). (**b**) In the periphery and in contact with liver tissue, there is an abundant fibrinous coating rich in neutrophils. The adjacent liver tissue shows moderate steatosis (Haematoxylin and Eosin stain, ×200).

**Figure 4:**
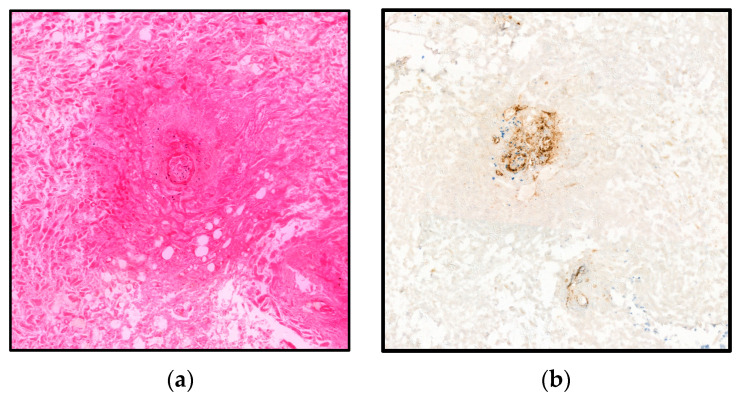
The vascular lesion in emphysematous hepatitis of our case report. (**a**) This image shows microthrombosis of small blood vessels within necrotic liver tissue g (Haematoxylin and Eosin stain, ×200). (**b**) This image shows the endothelial cells boarding the vascular lumen, small blood vessels and capillaries stained by the vascular marker CD34 (CD34, Immunohistochemistry ×200).

The histological examination of the surgical specimen showed necrotic hepatic tissue, a diffuse fibrinous coating associated with numerous neutrophils (Figure 3). The microscopical examination also revealed several images of microthrombosis (Figure 4).

The patient’s general condition improved progressively with the disappearance of the fever. Antibiotics were continued for a total course of three weeks without adaptation to the microbiological documentation. A follow-up CT scan 20 days after surgery showed a common postoperative collection in the resection site with no evidence of emphysema. The patient was discharged from the hospital 23 days after arrival.

### 2.2. Literature Review

We performed a bibliographic search on the PubMed, Cochrane, and Google Scholar websites of all English-published cases of emphysematous hepatitis from inception until December 2021, using the following keywords: emphysematous hepatitis, emphysematous liver abscess and emphysematous liver. We eliminated manuscripts concerning animals, duplicate cases, emphysematous involvement of an organ other than the liver (including emphysematous cholecystitis), manuscripts with no link to our subject, and reviews with no new case. We then excluded patients with pyogenic abscesses with airborne components, defined by the presence of fluid or the presence of an organised structure (rounded or oval delineation, presence of wall or septa). Key information about the articles was extracted into a predefined datasheet: study characteristics include author, year of publication, patient characteristics (age and comorbidities), laboratory values (PCT levels, WBC levels, CRP levels, AST/ALT, bilirubin), method of diagnosis and imaging results, microbiological documentation (blood culture and/or culture from a liver sample), patient’s management and outcome. We identified 14 cases in the literature (Table 1) [1,3,4,5,6,7,8,9,10,11,12,13,14,15].

## 3. Results and Discussion

**Figure 5:**
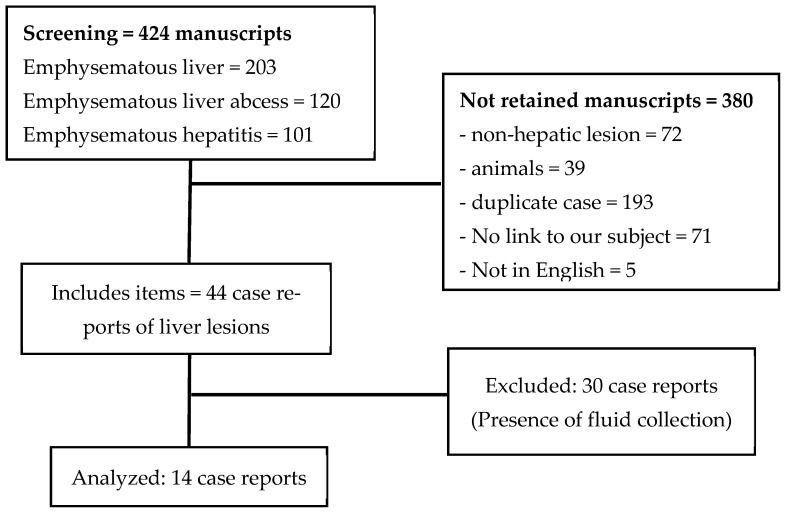
Flow chart of bibliographic search on PubMed, Cochrane and Google Scholar websites.

As described in the flow chart (Figure 5), of the 424 manuscripts selected from these research criteria, only 14 cases were retained as emphysematous hepatitis, half of them published between 2019 and 2021 [10,11,12,13,14]. Our case being the 15th, we compared the available data of the 14 patients (Table 1).

The mean age was 67 years (ranging from 38 to 82), with a female predominance (10 women/5 men). Eight patients had diabetes (53%), and four (27%) had a history of abdominal cancer (three cholangiocarcinoma and one pancreatic cancer) [5,8,9,11]. Two patients had no medical history [14,15].

The evolution of the disease was almost always fulminant. The average duration of symptoms before the consultation was 2.6 days. Ten patients (77%) developed hemodynamic failure within the 24 h following hospital admission, and 11 (73%) died within a mean time of 2 days (from 1 to 8 days).

Clinical symptoms were non-specific, with dysthermia, abdominal pain, and tenderness on palpation. Biological analyses showed neutrophil hyperleukocytosis, increased CRP, cytolysis predominantly with AST and increased total bilirubin (Table 2). Only one patient had a PCT measurement, which was 83.86 ng/mL. It would be interesting to assess whether PCT is more sensitive in emphysematous hepatitis, as observed in hepatobiliary infections [16]. The diagnosis was based on a CT scan, sometimes associated with an ultrasound exam. One patient presented with anaemia associated with *C. perfingens bacteraemia* (8), possibly toxaemic.

Patients were mostly bacteremic. The two most frequently identified microorganisms were *Clostridium perfingens* and *Klebsiella* spp. (five *pneumoniae* and one *oxytoca*) (Table 3). The other identified microorganisms were *Escherichia coli*, *Enterobacter cloacae*, *Streptococcus mutans*, *Enterococcus faecalis* and *Enterococcus faecium* [3,7,9,12,14].

Most patients received antibiotic therapy, but the details concerning the molecule were often not available. Management consisted of antibiotics alone for eight patients, and radiological drainage and antibiotics for three patients. Four patients received antibiotic therapy combined with surgical management within 48 h of hospital admission. This appears to be the main difference between survived and dead patients (Table 2). There are insufficient data to determine whether surgery should be performed systematically from the outset of management, or whether in low-risk patients, as in the case of biliary infections, percutaneous drainage followed by reassessment at 24–48 h is possible [17].

Patients with diabetes were more likely to have *Klebsiella*-related infection, with no other significant difference with non-diabetic patients. We did not observe any difference according to the causal microorganism *Clostridium* versus *Klebsiella*, given the small number of cases.

The pathophysiology of emphysematous hepatitis is poorly understood. Given the number of patients with unbalanced diabetes, one of the hypotheses put forward is a mechanism similar to emphysematous cystitis and pyelonephritis [2,18,19]. Thus, we can also hypothesise that emphysematous hepatitis is related to gas production by the use of an anaerobic pathway and bacterial fermentation, promoted by microcirculatory abnormalities associated with a reduction in the immunity of the host and a high tissue glycaemic index [18]. The most frequently isolated bacteria in this context are *Escherichia coli* and *Klebsiella pneumoniae* [19].

Nevertheless, part of the patients was not diabetic but had cancer. In these patients, the involvement of *Clostridium perfingens* with the appearance of anaerobic conditions following vascular remodelling related to cancer or in the aftermath of surgery [20] would probably be the alternative pathomechanism. These fast-growing bacteria produce numerous toxins, notably alpha toxin, which inhibits the maturation and migration of neutrophils, promoting the release of oxygen free radicals. The latter can be responsible for the vasoconstriction effect and platelet aggregation leading to the formation of secondary necrosis [21]. Moreover, perfingolysin O acts in synergy with alpha toxin [22] by forming pores, targeting the red blood cells and promoting the appearance of disseminated intravascular coagulation explaining the rapidity of the clinical picture [23]. Interestingly, we observed several images of vascular micro thrombosis within the surgical specimen, which can support this mechanism (Figure 4).

For all these reasons, clinicians should be alert to the possibility of *Clostridium perfingens* infection in the presence of a liver lesion with suggestive radiological aspects and non-specific clinical and laboratory findings. In this case, clinicians must rapidly introduce appropriate antibiotics. Metronidazole or Clindamycin would be the molecules of choice, combining with an antitoxin effect and rapid reduction of the bacterial load [24].

In our case, the responsible bacterium was *Klebsiella pneumoniae* with a hypermucosal phenotype. In the literature, we found only one case describing a liver abscess with gas production associated with the presence of a hypervirulent *Klebsiella* serotype K1 ST23. Unfortunately, we were unable to perform sequencing on the isolated strain in our patient, making it difficult to compare.

The histologic feature of emphysematous hepatitis was briefly described in a postmortem liver specimen obtained by autopsy [4]. The authors described clearly defined empty spaces covered by a patch of leukocytes without other associated changes. In our case, the histologic image is characterised by a space with an upper roof of completely necrotic liver tissue replaced by a fibrinous and haemorrhagic coating with numerous neutrophils. For the first time, we described the presence of several micro thrombosis within the necrotic tissue.

## 4. Conclusions

Emphysematous hepatitis is a rarely described infectious disease. We described the 15th case, and we reviewed the 14 previous ones reported in the literature. We also describe the associated histopathological changes. Our patient had a favourable outcome, probably due to prompt surgical management. Our manuscript underlines the need to perform biological and histopathological sampling to better understand the pathophysiology of this rare entity, which seems to be multiple. Thus, we believe that emphysematous hepatitis should be considered a syndrome rather than a simple disease.

## Figures and Tables

**Table 1:** Comparative table describing demographic characteristics (age, sex and country of patient), symptoms and clinical presentation, microbiological findings, associated management (other than antibiotic treatment), and outcome of the 14 cases with emphysematous hepatitis (excluding our case). M = Male, F = Female.

Author	Age (Year)	Sex	Underlying Disease	Symptoms	Location	Microbiology	Associated Treatment	Outcome
Blachar et al., 2002 (USA) [1]	43	F	Smoker, hyperlipidaemia, diabetes mellitus, short bowel syndrome for ischemic episode	Sepsis and multi-organ failure	Right hepatic lobe	*Klebsiella pneumoniae*	Percutaneous drainage	Died (72 h)
López Zárraga et al., 2006 (Chili) [4]	72	M	Renal lithiasis, a hip replacement, and type 2 diabetes mellitus	Abdominal pain and temperature at 35.2 °C	-	*Klebsiella oxytoca*	-	Died (8 h)
Létourneau-Guillon et al., 2010 (Canada) [5]	53	M	Left hepatectomy with hepaticojejunostomy for hilar cholangiocarcinoma	Chills since 36 h, fever and icterus	The right lobe of the liver, centred on segment VIII	*Enterobacter cloacae* *Clostridium Perfringens*	-	Died (36 h)
Lin et al., 2012 (Taiwan) [6]	59	F	Glucose intolerant	Two days of epigastric pain, tenderness in the upper right quadrant, temperature at 34.8 °C, blood pressure at 81/53 mmHg	-	*Klebsiella pneumoniae*	-	Died (20 h)
Chauhan et al., 2012 (India) [7]	77	M	Poorly controlled diabetes	Six Days of abdominal pain. Altered consciousness, fever and shock on the day of the presentation	Segments VI and VII of liver	-	Percutaneous drainage	Died (72 h)
Kim et al., 2012 (Korea) [8]	80	F	Hilar cholangiocarcinoma, endoscopic retrograde biliary stent, radiation therapy	Two days of severe right upper quadrant abdominal pain of 2 days	Superior segment of the right liver	*Clostridium perfringens* *Escherichia coli*	Percutaneous drainage	Died (72 h)
Nasa et al., 2017 (USA) [9]	73	F	Pancreatic cancer with hepatic and lung metastatic, Whipple operation, chemotherapy pulmonary embolism, chronic obstructive pulmonary disease, hypertension, chronic hepatitis C	Two days of abdominal pain and altered mental status	Right hepatic lobe	*Streptococcus mutans* *Enterococcus faecalis*	-	Died (24 h)
Ghosn et al., 2019 (Lebanon) [3]	38	F	Gestational diabetes mellitus Cholecystectomy, 2 C-sections, and ventral hernia repair	Two days of chills and fever, then sudden-onset abdominal pain and fever	Left hepatic lobe, mainly in segments II and III	*Escherichia coli* *Enterococcus faecium*	Rapid surgical debridement	Discharged after 13 days
H. Calderon 2020 (USA) [10]	80	F	Hypertension, diabetes mellitus type 2, chronic kidney disease stage 3	8 hours’ history of gradual onset upper abdominal pain and nausea	-	*Clostridium perfringens*	-	Died (16 h)
Azri et al., 2020 (France) [11]	75	F	Hilar cholangiocarcinoma, biliary endoscopic drainage with stent and portal embolisation, cyberknife stereotactic radiotherapy	Left upper abdominal pain without tenderness and fever	Left hepatic	*Klebsiella pneumoniae* *Escherichia coli* *Enterococcus faecalis* *Clostridium perfringens* *Aeromonas ichtiosmia*	-	Died (<24 h)
Miranda et al., 2020 (Portugal) [12]	75	M	Hypertension, gastroesophageal reflux, and moderate alcohol consumption	Two days of fever, cough with mucopurulent sputum, and abdominal pain	Right hepatic lobe	*Escherichia coli*	-	Died (72 h)
Boffil et al., 2021 (Spain) [13]	74	M	Diabetes, chronic pancreatitis, and biliary strictures	One day history of fever and confusion, tenderness in the upper right quadrant	Right hepatic lobe	*Klebsiella pneumoniae*	Percutaneous drainage	Died (8 days)
Estébanez-Ferrero et al., (Spain) [14]	67	F	-	Abdominal pain and vomiting	Right hepatic lobe	*Escherichia coli*	Rapid surgical debridement	No relapse after 3 years
Ramalho et al., 2017 (Portugal) [15]	78	F	-	Upper right quadrant pain, nausea and fever	Left hepatic lobe with spontaneous rupture to the peritoneum	*Enterococcus faecium*	Left hepatic lobe lobectomy	Successfully treated

**Table 2:** Demographic characteristics, clinical and paraclinical presentations, microbiological findings, management, and evolution of the 15 patients with emphysematous hepatitis (including our case).

	n	Results
Demographics		
The average age in years (range)	15	67 (38–82)
Sex ratio (M/F)	15	0.5
Past medical history		
Diabetes—n (%)	15	8 (53)
Digestive surgery < 6 months—n (%)	15	4 (27)
Digestive cancer—n (%)	15	4 (27)
Clinical presentation		
Number of days from symptom onset to consultation—average (range)	10	2.6 (1–6)
Hemodynamic failure within 24 h—n (%)	13	10 (77)
Temperature > 38° or <36 °C—n (%)	14	10 (79)
Confusion—n (%)	10	5 (50)
Abdominal pain—n (%)	14	12 (86)
Sensitivity—n (%)	11	8 (73)
Paraclinical presentation		
Average leukocytes—G/L (range)	11	22.7 (5.7–54.9)
Mean CRP—mg/L (range)	5	295 (13.5–493.6)
Mean AST—IU/L (range)	11	2355 (99–10 920)
Mean total bilirubin—µmol/L (range)	7	145.3 (23.8–465)
Presence of necrosis on CT—n (%)	14	4 (29)
Management		
Antibiotics	15	15 (100)
Antibiotics alone—n (%)	15	8 (53)
Radiological drainage without surgery—n (%)	15	3 (20)
Surgery—n (%)	15	4 (27)
Evolution		
Deaths	15	11 (73)
Number of days between consultation and death—Average (range)	11	2 (1–8)
Microbiology		
*Clostridium perfingens*—n (%)	14	4 (29)
*Klebsiella* spp.—n (%)	14	6 (43)
Bacteremia—n (%)	15	11 (73)

**Table 3:** Comparative table of patients with emphysematous hepatitis according to outcome (top), presence of diabetes (middle), and type of bacteria by microbiological analysis (down).

**Variable**	**Alive (n = 4)**	**Dead (n = 11)**
Cancer—n (%)	0 (0)	4 (36)
Surgery—n (%)	0 (0)	4 (36)
Diabetes—n (%)	2 (50)	6 (55)
*Klebsiella* spp.—n (%)	1 (25)	5 (45)
*Clostridium perfingens*—n (%)	0 (0)	4 (36)
Antibiotics alone—n (%)	0 (0)	8 (73)
Radiological drainage alone—n (%)	0 (0)	3 (24)
Surgery—n (%)	3 (100)	0 (0)
**Variable**	**Diabetics (n = 8)**	**Non-Diabetics (n = 6)**
Cancer—n (%)	0 (0)	4 (67)
Surgery—n (%)	1 (13)	3 (50)
*Klebsiella* spp.—n (%)	5 (71)	1 (17)
*Clostridium perfingens*—n (%)	1 (13)	3 (50)
Deaths—n (%)	6 (75)	5 (83)
**Variable**	***Clostridium* (n = 3)**	***Klebsiella* spp. (n = 4)**
Cancer—n (%)	2 (66)	1 (25)
Surgery—n (%)	2 (66)	1 (25)
Diabetes—n (%)	1 (33)	3 (75)
Presence of necrosis on CT—n (%)	1 (33)	2 (50)
Deaths—n (%)	3 (100)	3 (75)

## Data Availability

The data used and analysed during the current study are available in the manuscript text, figures and tables. Further information could be obtained from the corresponding author upon reasonable request.
